# Toll-like receptors in atopic dermatitis: pathogenesis and therapeutic implications

**DOI:** 10.1016/j.heliyon.2025.e42226

**Published:** 2025-01-31

**Authors:** Ahmad Vafaeian, Fateme Rajabi, Nima Rezaei

**Affiliations:** a Universal Scientific Education and Research Network (USERN), Tehran, Iran; b Autoimmune Bullous Diseases Research Center, Tehran University of Medical Sciences, Tehran, Iran; c Center for Research & Training in Skin Diseases & Leprosy, Tehran University of Medical Sciences, Tehran, Iran; d Network of Immunity in Infection, Malignancy and Autoimmunity (NIIMA), Universal Scientific Education and Research Network (USERN), Sheffield, UK; e Research Center for Immunodeficiencies, Children's Medical Center, Tehran University of Medical Sciences, Tehran, Iran; f Department of Immunology, School of Medicine, Tehran University of Medical Sciences, Tehran, Iran

**Keywords:** Atopic dermatitis, Itch, Pathogenesis, Toll-like receptors, Innate immunity

## Abstract

Toll-like receptors (TLR), the key players of the innate immune system, contribute to the pathogenesis of atopic dermatitis (AD) through multiple pathways. TLRs play a crucial role in delaying barrier repair, promoting Th2-mediated dermatitis, shifting the response toward Th1 in the chronic phase, and contributing to the establishment of the itch-scratch cycle, as well as mediating the effects of UV radiation. The dysregulation of proinflammatory and immunomodulatory effects of TLRs can be attributed to their ligand structures, receptor heterodimerization, the relative frequency of each TLR, interactions with other receptors/signalling pathways, cytokine milieu, and genetic polymorphisms. Current AD treatments like vitamin-D analogs, tacrolimus, and cyclosporine partially work through TLR modulation. Direct TLR stimulation using different compounds has shown therapeutic benefits in preclinical studies. However, significant challenges exist, including off-target effects due to ubiquitous TLR expression and complex roles in immune responses. Future directions include CRISPR-based gene editing to understand TLR functions, development of specific TLR modulators for targeted therapy, and machine learning applications to predict drug responses and identify novel ligands. Patient heterogeneity, including the presence or absence of polymorphisms, variations in TLR expression levels, and differences in immune responses, underscores the need for personalized therapeutic approaches.

## 1 Introduction

### 1.1 Atopic dermatitis

**Fig. 1:**
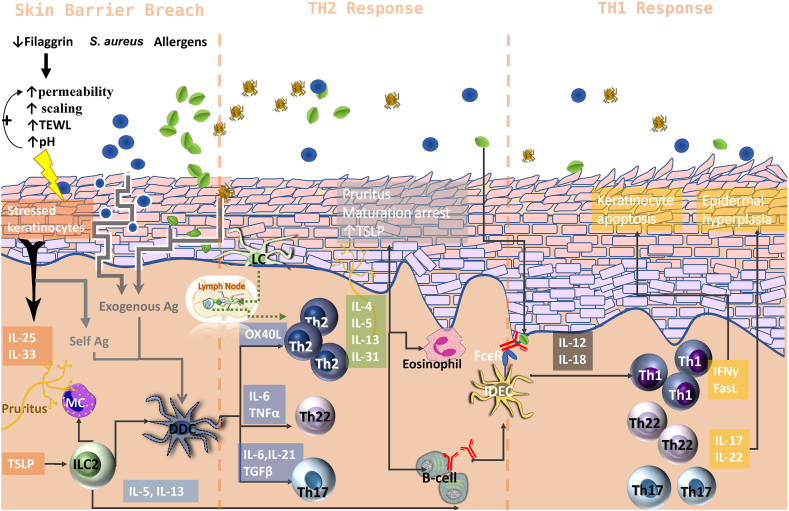
The three sequential phases of the pathogenesis of atopic dermatitis. TEWL, transepidermal water loss; MC, mast cells; ILC, innate lymphoid cells; dDC, dermal dendritic cells; IDEC, inflammatory dendritic epidermal cells; Th, T-helper; LC, Langerhans cells; TSLP, thymic stromal lymphopoietin; IL, interleukin; Ag, antigen; TNFα, tumor necrosis factor-alpha; TGFβ, transforming growth factor-beta; IFNα, interferon-alpha; FcεR, Fc epsilon receptor.

Atopic dermatitis (AD) is a chronic inflammatory skin disease affecting mostly children [1]. The disease is characterized by pruritus, xerosis, erythema, and scaling and has a complex multistep course with both environmental and genetic factors playing a major role. AD progresses through three sequential phases of dermatitis with minimal inflammation, acute T-helper 2 (Th2) mediated dermatitis, and chronic Th1-mediated dermatitis [2] (Fig. 1). Occasionally, the inflammation progresses further to IgE-associated or auto-allergic atopic dermatitis.

In the first phase, epidermal barrier abnormalities, particularly the abnormalities in stratum corneum differentiation, are the main issue [3]. This is a consistent feature apparent in both lesional and non-lesional skin that would allow the exogenous irritants, antigens, and microorganisms to gain access to the deeper layers of the epidermis and engage the immune system [[4], [5], [6], [7], [8], [9], [10], [11]]. Filaggrin deficiency also leads to xerosis and elevated pH levels, which further compromise barrier permeability and promote increased bacterial colonization [5].

The loss of skin integrity allows the influx of exogenous antigens and imposes stress on the keratinocytes, increasing the apoptosis rate and releasing self-antigens [4]. These epicutaneous and self-antigens are uptaken by the Langerhans cells (LC) and dermal dendritic cells (dDC) that migrate to lymph nodes and induce immunity [12]. The stressed keratinocytes also release proinflammatory molecules such as thymic-stromal-lymphopoietin (TSLP), interleukin-1β (IL-1β), IL-25, and IL-33 [[13], [14], [15]]. TSLP activates type-2 innate lymphoid cells (ILC), which in turn endorses Th2 polarization and B-cell maturation by producing IL-5 and IL-13 [16]. TSLP also promotes the expression of the OX40 ligand on dDCs, which, through interaction with its receptor on naive T-cells, conveys a survival signal and endorses the differentiation of Th2 cells [17].

The predominance of the Th2 -cells marks the second step in the course of AD. These cells produce massive amounts of IL-4, IL-5, IL-13, and IL-31 that are capable of creating a feedforward loop by suppressing the maturation of the stratum corneum, inciting the expression of TSLP, and triggering the itch and scratch cycle by stimulating cutaneous nerves [[18], [19], [20]].

Though Th2 cells are the dominant cells in the acute phase of AD, dDC and LCs also contribute to the differentiation of Th22 and Th17 cells through excretion of IL-6/tumor necrosis factor α (TNFα) and IL-6/IL-21/Transforming growth factor β (TGFβ), respectively [21,22]. These cells produce IL-22 and IL-17 that can suppress the differentiation of keratinocytes and contribute to epidermal hyperplasia [23].

A subset of inflammatory DCs that express IgE receptors (FcεRI and II) and have been described in atopic skin are activated upon encountering percutaneous antigens, which, along with the efficient antigen presentation, these activated cells release IL-12 and IL-18 that polarize naïve T-cells towards the Th1 phenotype [24,25]. The chronic phase of AD is marked by the increase in Th1 cells, interferon-γ (IFNγ), and Fas/Fas ligand-mediated keratinocyte apoptosis, although the Th2 cells are still dominant [26,27]. Other cell lines such as macrophages, mast cells, and basophils are also recruited into the atopic skin by the lesional chemokine milieu. These cells contribute to the pathogenesis of AD by perpetuating the inflammation and releasing pruritogens [28].

### 1.2 TLRs: Function and historical background

**Fig. 2:**
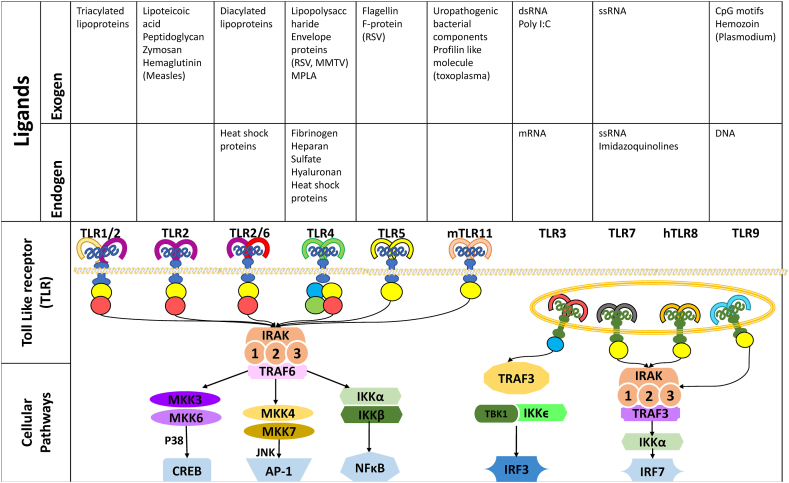
Toll-like receptors, their endogenous and exogenous ligands, their cellular signaling pathways. RSV, respiratory syncytial virus; MMTV, mouse mammary tumor-like virus; MPLA, Monophosphoryl lipid A; IRAK, IL-1 Receptor-Associated Kinase; TRAF, Tumor necrosis factor receptor–associated factor; MKK, mitogen-activated protein kinase; IKK, IκB kinase; CREB, cAMP Response Element-Binding Protein; AP-1, Activator protein-1; IRF, interferon regulatory factor; NF-κB, nuclear factor kappa-light-chain-enhancer of activated B cell.

Toll-like receptors (TLRs) are a subgroup of pattern recognition receptors capable of detecting environmental pathogens by their preserved molecular signatures. Upon stimulation, TLRs can trigger different inflammatory pathways that result in the non-specific elimination of the pathogens. In humans, ten distinct types of TLRs have been identified, each recognized by specific ligands and associated with unique downstream signaling pathways (Fig. 2) [[29], [30], [31]].

The understanding of TLRs has evolved significantly since their discovery, marking several key historical milestones. Before the discovery of TLRs, innate immunity was viewed as a simple and unrefined component of the immune system. It was primarily thought to serve the function of initiating the more advanced adaptive immune response and triggering systemic reactions [32]. TLRs were first identified as pattern recognition receptors (PRRs) in the 1990s, playing a vital role in the innate immune system by recognizing pathogen-associated molecular patterns (PAMPs) and danger-associated molecular patterns (DAMPs) while serving as a bridge between the innate and adaptive immune systems [33]. The identification of specific ligands, such as lipopolysaccharides (LPS) for TLR4, was a major advancement that linked TLRs to bacterial recognition and immune responses [34]. Subsequent advances using TLR-deficient mice revealed that the mammalian TLR family comprises over 12 members, with TLR1–TLR9 conserved between humans and mice, TLR10 inactive in mice due to a retroviral insertion, and TLR11, TLR12, and TLR13 absent in humans, while also identifying specific ligands for each TLR [35]. Meanwhile, the signaling pathways of TLRs were elucidated, particularly the MyD88-dependent and TRIF-dependent pathways, which control the production of proinflammatory cytokines and type I interferons, respectively [36]. The 2011 Nobel Prize in Physiology or Medicine was partially awarded to Bruce A. Beutler and Jules A. Hoffmann for their discoveries regarding the role of TLRs [34,37]. These discoveries paved the way for understanding TLR involvement in a range of immune responses, including vaccination, as well as in various diseases, such as autoimmune and inflammatory conditions [32].

This review addresses a critical need in dermatology and immunology by integrating current knowledge on the role of TLRs in AD. Current knowledge of TLRs in AD leaves gaps in fully understanding their mechanisms of action, contributions to immune dysregulation, aberrant Th1/Th2 responses, skin barrier dysfunction, and potential therapeutic implications. Given recent therapeutic advancements targeting TLR pathways [38,39], it is timely to consolidate existing research and highlight emerging insights. Additionally, there are inconsistencies in the literature (Table 1) that this review aims to clarify. It will also explore the relevance of TLR signaling in AD and provide new perspectives to inform future therapeutic and research directions.

**Table 1:** – Toll-like receptor (TLR) expression levels in atopic dermatitis.

Status	TLR	Tissue/Cell	Details	References
**Upregulated**	TLR2	Monocytes	Higher at exacerbation period, Associated with AD clinical severity	[45,84]
	TLR2/4	Whole lesional epidermis	Tacrolimus may directly or indirectly down-regulate TLR2 and TLR4 expression in KC	[207]
	TLR4	Epithelial membranes	An HMGB1-TLR4-NF-κB signaling pathway is activated in atopic dermatitis.	[123]
	TLR2/4	Peripheral Monocytes	Higher CD14/TLR components	[208]
	TLR2/4/5	PMN		[209]
	TLR2	Nucleated epidermis	Increases towards lower spinous and basal layers.	[210]
	TLR4	Nucleated epidermis	Increases towards the upper spinous and the granular layers	[210]
	TLR3	Stratum corneum	Correlates with the intensity scores of the disease	[211]
**Downregulated**	TLR4	Non-lesional skin	Lower gene expression was associated with greater AD severity	[62]
	TLR2/4/9	Keratinocytes		[46]
	TLR2	Keratinocytes	TLR2 expression inversely correlated with transepidermal water loss (TEWL) in the study by Kuo et al.	[49,212,213]‘
	TLR2	Macrophages/Monocytes		[214,215]
	TLR5/9	Dendritic cells		[51]
	TLR9	Cord blood	Higher expression at birth was associated with a lower risk of subsequent AD	[152]
	TLR2/4	Macrophages	NC/Nga mice	[216]
	TLR2	PBMC regulatory T-cells		[160]
	TLR2/4	CD4^+^ T cells	Impaired in patients with AD bearing R753Q SNP, Increases with LTA	[217]
	TLR2	Hair follicles keratinocytes		[55]
	TLR1/6	Monocytes		[146]
**No alteration**	TLR2/4	Monocytes		[215]
	TLR2	Hair follicles keratinocytes		[55]
	TLR2	Monocytes		[146]
	TLR1/6	Macrophages		[214]
	TLR6	Peripheral monocytes		[45]

### 1.3 Search methods

In the initial stage, we used the keywords “TLR,” “Atopic Dermatitis,” and “TLR and Atopic Dermatitis” in the PubMed database. Additionally, we examined the references cited in these articles to identify further relevant sources. For each subsection, other search queries were employed to gather a sufficient body of evidence, enabling us to provide well-supported reasoning with minimal logical gaps regarding the mechanism of action of TLRs in AD pathogenesis. To maintain comprehensiveness, no filters were applied regarding publication date, study type, or study population. References were selected from peer-reviewed publications, and for experimental comparative studies, only those results with a P-value <0.05 were considered. Study methods were thoroughly verified to ensure the methodological rigor and validity of the findings. We utilized a narrative approach to summarize and integrate findings from the included studies. This method was chosen due to the heterogeneity of the studies in terms of design, populations, cell lineages, and outcomes. Furthermore, the findings were organized thematically.

## 2 Role of TLRs in atopic dermatitis pathogenesis

The innate immune system including TLRs is the first responder in the event of a barrier breach, the most consistent feature of AD. Higher TLR expressions on the internally positioned cells may be a result of repeated exposure to agents and allergens leaked through the defective barrier [40,41] (Table 1).

### 2.1 Impaired TLR function impedes pathogen clearance in AD

Ideally, TLRs activation should result in the elimination of the pathogens with minimal inflammation but patients with AD, they demonstrate a decreased ability in pathogen clearance and a tendency toward an exaggerated inflammation [[42], [43], [44]]. It has been shown that staphylococcal enterotoxin B elevates TLR6 expression on peripheral monocytes in patients with AD but not in the control group [45].

Numerous studies have provided examples of impaired TLR function in atopic individuals against infections, which may result from TLR genetic polymorphisms, desensitization of receptors due to prolonged exposure to ligands, the inhibitory effect of other cellular receptors due to competitive interaction over transcription factors, or post-transcriptional defects [[46], [47], [48], [49], [50], [51], [52]] (Table 1).

Patients with atopy also show impaired TLR2-stimulated cytokine and chemokine release from both immune cells and keratinocytes [47,49,[52], [53], [54], [55]]. Upon TLR2 stimulation, dendritic cells and CD14dim FcεRI-high subpopulation of monocytes from patients with AD show reduced maturation and cytokine production [51,53,56]. Defective pathogen clearance is clinically translated into a higher rate of disseminated cutaneous viral infections such as eczema herpeticum and the abundance of *Staphylococcus aureus* (SA) colonization in atopic skin [[57], [58], [59], [60]]. This has been attributed to both the lower expression of CD14 that facilitates the attachment of the ligands to the TLR2/1 complex and the higher expression of FceRI+, which may oppose TLR2-mediated responses [53,54,61]. Reduced expression of TLR4 on a variety of cells from patients with AD is associated with more severe eczema vaccinatum [62]. It has been shown that molecules produced by the vaccinia virus inhibit TLR3 downstream pathways [[63], [64], [65]]. TLRs can also mediate host defense through the expression of antimicrobial peptides (AMP), such as human-beta-defensin 2 (HBD-2) and LL-37 [[66], [67], [68], [69], [70]]. Vaccinia virus promotes LL37 production in a TLR3-dependent manner, which is inhibited by the Th2 cytokine milieu of AD [70,71].

SA colonization in atopic skin can further induce immunosuppression and secure its dominance by activating the myeloid-derived suppressor cells (MDSCs) in a TLR-dependent manner. The induction of MDSCs which inhibits T-cells occurs only upon TLR2/6 activation [50,72]. TLR2/6 complex has more affinity for diacylated lipoproteins which are more abundant in the post-exponential phase of growth of SA due to resource limitations [[57], [58], [59], [60],^73^,^74^].

### 2.2 TLRs promote detrimental Th2 responses in AD

TLRs are involved in the promotion of Th2 response, as SA-activated TLR2 interferes with the IFNGR/JAK/STAT1 pathway which results in the downregulation of the Th1 cell–recruiting chemokines [60,75,76]. Furthermore, SA-associated peptidoglycan-embedded ligands stimulate the TLR2 to produce IL-10, which in turn reduces the Th1/Th17 responses [77,78].

TLR3 alone or in synergism with type I IFNs and Th2 cytokines (IL-4 and IL-13) induces the expression of TSLP in epithelial cells. TSLP activates the ILC2 cells, which in turn drive the immune response towards Th2, further disrupting the skin's ability to defeat viral infections [60,69,[79], [80], [81], [82], [83]]. Also, there is a positive correlation between the frequency of TLR2+ monocytes and serum IL-4 levels during AD exacerbations [84]. Furthermore, type I IFNs abundant during viral infections can increase both TSLP and TLR3 expression in the keratinocytes [79,85,86]. This results in a vicious cycle in which TSLP and Th2 cytokines reinforce each other [79]. Upon TLR3 stimulation, keratinocytes produce IL-33 and IL-31 [[87], [88], [89], [90], [91], [92]]. The ΔNp63 isoform of p63, which is also a TLR3 stimulant, upregulates IL-33 and IL-31 as its transcriptional targets [[88], [89], [90]]. IL-33 activates the type 2 ILCs that subsequently promote a Th2-dominated cytokine milieu.

Activated basophils also promote the Th2 response via other TLR-dependent pathways [93,94]. Patients with AD have a higher basophil activation ratio following TLR4 stimulation [95]. It has been shown that TLR2-expressing basophils and dermal fibroblasts mediate the SA-associated exacerbation of AD [96]. In general, the peripheral blood mononuclear cells, monocytes, and dendritic cells of patients with AD release higher amounts of Th2 cytokines and lower amounts of Th1 cytokines upon TLR2 and TLR4 activation [54,55,[97], [98], [99], [100], [101]].

Contrariwise, impaired TLR signaling benefits the Th2 responses as it has been shown that TLR2 and 4 deficient mice failed to upregulate IFN-γ expression but keeped the local expression of IL-4 intact [97,[102], [103], [104]]. Allergen challenge in TLR4 deficient mice instigates severe dermatitis with an exaggerated influx of inflammatory cells and high levels of Th2 cytokines, TNF-α, and TSLP without conversion to a Th1 mediated inflammation [102,104].

### 2.3 TLRs drive Th1-Mediated chronic inflammation in AD

Besides Th2 cells, in the chronic phase of AD, Th1 cells and their associated cytokines contribute to prolonged and detrimental inflammation [76]. Multiple studies have demonstrated the essential role of TLRs in shifting the milieu towards Th1. In the IL-4-rich environment of acute AD, stimulation of TLR2, with SA-derived lipoteichoic acid results in upregulation of IL-12/IL-10 ratio and IFN-γ that provoke chronic inflammation [105,106]. Monocytes from patients with AD carrying the TLR2-R753Q risk allele produce higher levels of IL‐12 in comparison to the wild-type [100]. TLR2 stimulation also increases IL-17A produced by monocytes whose serum level correlates with AD severity [54,84,107]. Furthermore, TLR2^−/−^ mice do not develop chronic Th1-mediated features of AD following epicutaneous sensitization [97].

### 2.4 TLRs exacerbate pruritus and impair skin barrier repair in AD

TLRs are involved in the peripheral and central neurological pathways of pruritus [[108], [109], [110]]. TLR 3 and 7 ligands trigger action potentials in dorsal root ganglion (DRG) neurons and provoke scratching [108,109]. Both histamine-dependent and histamine-independent itch/scratch responses are reduced in TLR3^−/−^ mice, but only histamine-independent pruritus is affected in TLR7^−/−^ mice [108,109]. The production of nerve growth factor (NGF) as a peripheral mediator of pruritus seen in wild-type mice following the induction of dry skin-resembling lesions is also absent in TLR3^−/−^ mice [109]. TLR3 also affects the central perception of pruritus as it increases the frequency of short-term excitations and affects long-term synaptic plasticity [109].

Atopic skin demonstrates an exaggerated inflammatory response to subtle physical insults such as itch/scratch that can liberate several danger molecules, which are recognized by TLR2 and TLR4. TLR2 promotes IL-31 secretion and thus further worsens pruritus and establishes a vicious cycle [48]. In AD mice models, tape-stripping, as a substitute for pruritus, releases endogenous TLR4 ligands that contribute to the production of IL23p19 which in turn primes IL-23R expressing DCs to produce more IL-23. These cells, in turn, polarize naïve T-cells to drive an IL-22 response to percutaneous antigens and cause an excessive inflammatory response [111,112].

The itch-scratch cycle is further perpetuated as patients with AD often demonstrate delayed barrier repair responses in which TLRs are involved [62,113]. TLR deficiency delays barrier repair and increases trans-epidermal water loss (TEWL), and the application of TLR agonists can accelerate barrier healing [[114], [115], [116]]. Tissue damage liberates endogenous dsRNA, hyaluronic acid, heparin sulfate, and HSP that activate TLRs, promoting the release of cytokines such as IL-6 and IL-1β which are essential for initiating tissue repair [49,113,[117], [118], [119]]. TLR2-and TLR4-deficient AD mice models show increased barrier dysfunction [49,97,114]. TLR2 upregulation of skin barrier components is severely impaired in keratinocytes of patients with AD where TLR2 expression level is low compared to healthy individuals [49]. TLR3 upregulates stratum corneum components and recruits neutrophils and alternatively-activated healing macrophages [[119], [120], [121]]. The secretion of IL-6 and TNF-α, two important cytokines for skin repair, is impaired following TLR2 stimulation in keratinocytes from AD subjects [49,55]. Alternatively, TLR-mediated exaggerated inflammation can also have negative effects on all aspects of repair mechanisms [[122], [123], [124]].

### 2.5 TLRs mediate the dual effects of UV radiation on AD: repair and exacerbation

Ultraviolet radiation (UV), specifically UVB exposure, has paradoxical effects on AD. It is considered a second-line treatment option for severe AD, but it can also exacerbate atopic lesions [125,126]. TLRs can mediate both of these effects. TLR3 downstream signaling pathway is necessary for tissue repair after UV damage [[127], [128], [129]]. UVB radiation promotes a structural change, known as stem-loops, in noncoding RNAs released from sun-damaged keratinocytes, such as U1 spliceosome RNAs, which act as endogenous ligands for TLR3 and upregulate the expression of genes necessary for skin barrier repair and cytokines such as IL-6 and TNF-α [127,128,130,]]. Radiation-induced immunosuppression is also partly mediated through TLRs as UVB-induced suppression of contact hypersensitivity was present in wild-type mice but not TLR3^−/−^ or TLR4^−/−^ mice [127,131]. TLR4 pathways promote CD4^+^CD25+Foxp3+ T-reg-cells, and TLR3 induces the expression of immunomodulatory cytokines [131,132].

TLR3 contributes to the exacerbation of AD following UV exposure by meddling with the expression of the p53 family of transcription factors [[133], [134], [135], [136]]. Two factors implicated in epidermal development, ΔNp63 and ΔNp73, are affected by TLR3, leading to an upregulation of the TSLP pathway [88,89,137]. The former is downregulated, and the latter is unregulated upon TLR3 stimulation. These factors are implicated in healing and long-term differentiation toward a state favorable for AD.

### 2.6 TLR interactions with other receptors affect AD

Interactions with other macromolecules and receptors such as the adaptor Toll-interacting protein (Tollip), CD14, CD36, nucleotide-binding oligomerization domain-containing protein 2 (NOD2), and T-cell receptor (TCR) can also affect the TLR responses and the risk of developing AD [138,139]. CD36, in addition to CD14, facilitates the binding of diacylated lipoproteins to the TLR2/6 complex [61,140]. The CD36 gene is upregulated in patients with AD, and the impaired interaction of AD-associated TLR‐2 R753Q risk allele with CD36 might contribute to the higher susceptibility to SA in these individuals [141]. Dual NOD2/TLR-activated DCs by peptidoglycan moieties display significantly enhanced IL-12 and IL-23 production compared to those only stimulated with TLR ligands [142]. It has been shown that NOD2/TLR2-mediated exacerbation of AD can be through the activation of basophils interacting with dermal fibroblasts [96]. The TCR/TLR2 interaction also accentuates the inflammatory response in AD mice models [143].

Coordinated interactions between TLRs and FcɛRI also contribute to the skewed Th2 response [144]. FcɛRI is abundant in AD, and exposure to allergens, plasma IgE levels, and TLR2 stimulation can further increase its expression [54,56,145,146]. Prolonged bacterial colonization on atopic skin leads to higher IgE production and more interaction between FcɛRI and TLR2 [147]. Simultaneous activation of FcɛRI and TLR9 on plasmacytoid dendritic cells (PDC) suppresses the production of type-1 IFNs and increases the release of IL-10 in an autocrine manner, promoting PDC apoptosis. Lower PDCs favor the Th2 milieu and impair pathogen clearance [148]. In AD, upon allergen stimulation, the crosstalk between TLRs and FcɛRI, precisely TLR 2 and 4, promotes the release of IL‐4, IL‐8, IL‐13, and RANTES from basophils [54,149].

### 2.7 Polymorphisms in TLRs contribute to AD pathogenesis

Polymorphisms may either compromise the ability of TLRs to combat the pathogens or exaggerate the response and thus contribute to AD pathogenesis. SNPs in TLR1, TLR2, TLR4, TLR6, TLR9, and TLR10 have been implicated in AD, with details summarized in Table 2.

**Table 2:** Single nucleotide polymorphisms (SNP) associated with atopic dermatitis.

SNP ID	Alleles (Peptide)	Location	Population	Details	References
TLR2 rs5743708	c.2258 G>A (p.R753Q)	Exon	Italy	Associated with the incidence and the severity of AD. Higher serum IgE levels. Higher IL-4 and IL-10 serum levels and Lower serum INF-γ levels. Monocytes secrete lower amounts of IL-8. Lower levels of expression	[52,100,103,147,[218], [219], [220]]
			Russia		
			Germany		
TLR2 rs4696480	c.16934 A>T	Promotor	Italy	An allele is associated with the severity of the disease. Higher transcriptional activity of T allele	[219,221,222]
			Poland		
			Germany		
TLR4 rs4986790	c.896 A>G (p.D299G)	Exon	Italy	Associated with severe AD. Higher serum levels of IL-4 and IL-10. Lower serum INF-γ levels. Increased susceptibility to acute viral respiratory infections.	[103,219,223]
			Russia		
			Ukraine		
TLR4 rs2770150	c.-3612 T>C	Promotor	Netherlands	Risk allele: T	[173]
TLR4 rs1927911	c.8595 T>C	Intron	Europe and Canada	Associated with AD	[224]
TLR6 rs5743810	c.745C>T (p.P249S)	Exon	Finland	Risk allele: C	[225]
TLR6 rs5743794	c.-64-1569C>T	Intron	Ukraine	Risk allele: C	[226]
TLR1 rs5743618	c.1805 G>T (p.S602I)	Exon	Finland	G allele associated with AD during the first 6 years of life	[225]
TLR1 rs5743571	c.-160 + 438C>T	Intron	Ukraine	Risk allele: C	[226]
TLR1 rs5743604	c.-67-766 A>G	Intron	Ukraine	Risk allele: A	[226]
TLR9 rs5743836	c.-1237T>C	Promoter	Germany	Increased promoter activity of T allele associated with AD	[156]
TLR10 rs11466617	c.-62-3198 T>C	Intron	Ukraine	Risk allele: T	[226]

The most studied SNP associated with AD is TLR2 R753Q. Patients with this variant have lower serum IFN-γ levels and higher IgE and IL-4 levels [103,147]. Their monocytes secrete lower amounts of IL-8, which compromises the ability of patients to confront pathogens [52]. Conversely, macrophages with the SNP secrete more IL-12, thereby contributing to harmful inflammation [100]. The underlying mechanisms of the impacts of R753Q SNP on TLR2 function remain to be elucidated, but impaired TLR6 heterodimerization, tyrosine phosphorylation and recruitment Mal and MyD88 have been proposed [150,151]. In fact, the replacement of Arginine, a positive-charged amino acid, with Glutamine, a neutral amino acid, results in a decrease in the net positive charge of the DD loop [150]. Altered electrostatic properties of the TIR domain affect heterodimerization and the recruitment of MyD88 ^150^. Altogether, polymorphisms may lead to dysregulated TLR response and, thus, a higher incidence of AD or a more severe form of the disease.

There are paradoxical results on the effect of TLR9 in patients with AD. Children with higher expression of TLR9 at birth have a lower risk of subsequent AD [152]. Also, in both canine and mice AD models, transdermal application of TLR9 ligand ameliorates dermatitis and pruritus and improves cytokine milieu [[153], [154], [155]]. Conversely, a polymorphism in the TLR9 gene with increased promoter activity confers a higher risk for AD and SA strains from the skin of patients with AD, but not that of normal patients, promotes an IL-1α fueled inflammation through interaction with TLR9 [[155], [156], [157]].

### 2.8 TLRs mediate environmental effects on immune tolerance in AD

Individuals with atopy also demonstrate an impaired immunomodulatory response, allowing infectious agents to trigger an exaggerated inflammatory response through TLR-mediated suppression of IL-10 secretion and regulatory cell maturation, which play a significant role [50,69,158]. TLR2 and 4-expressing regulatory T-cells are less frequent in patients with AD compared to healthy individuals [159,160]. Defective B-reg cells from patients with AD show abnormal Signal Transducer And Activator Of Transcription 3 (STAT3) signaling induced by TLR [161]. Also, Decreased TLR4-mediated IL-10 secretion in neonates is associated with a higher risk of AD [107,161,162]. TLR4 stimulation by Poly-γ-glutamic acid (γPGA), through the TLR4/DC/IL12 axis, can promote anti-inflammatory effects by increasing the apoptosis of the basophils [158].

The role of environmental factors in impaired pathogen clearance in patients with atopy is explained by the hygiene hypothesis [163]. Guided by the exposure to the ligands during the first months of life, TLR responses mature the adaptive immunity from a Th2-skewed to a more balanced Th1 response [162]. Both skin and gastrointestinal microbiome can provide this ligand exposure [164]. Recognition of nonpathogenic bacteria such as *Staphylococcus epidermidis* (SE) and *Vitroscillia filiformis* by TLR2 leads to the production of IL-10, AMPs, inhibition of TLR3 inflammatory responses, and induction of IL-10+ suppressive DC and regulatory T-cells (T-regs) [68,106,118,165,166].

The abundance of gastrointestinal commensals such as Ruminococcaceae and Proteobacteria in one-week-old infants reduces the risk of developing AD by lowering the TLR2-induced IL-6/TNF-α and TLR4-induced TNF‐α secretions, respectively [167]. Pre- and postnatal intake of probiotics such as *Lactobacillus* and *Bifidobacterium* reduce the overall risk of subsequent AD development [168,169]. *Lactobacillus* exerts its immunomodulatory effects through TLR2, 4, and 9 stimulation, whereas TLR2 mediates the effects of *Bifidobacterium* [[170], [171], [172]]. Indeed, carriers of certain TLR gene polymorphisms show more prominent risk reduction with probiotic administration, *E. coli* colonization, and animal exposure [152,172,173].

## 3 Therapeutic implications and challenges of TLRs in AD

TLRs have emerged as promising targets for therapeutic interventions across a spectrum of diseases due to their crucial role in innate immunity and inflammation. TLR2 inhibition was found to prevent ischemic tissue injury in animal models [32]. It has been shown that TLR signaling in B cells is essential for generating effective antibody responses to vaccination, including BCG [174,175]. Imiquimod is a topical immune response modifier that acts as a TLR7 agonist and is used to treat genital warts, Bowen's disease, superficial basal cell carcinoma, and actinic keratosis [176,177]. TLR7 and TLR9 are implicated in the pathogenesis of systemic lupus erythematosus, and targeting these receptors or their downstream pathways has been shown to be a potential therapeutic approach [178]. Preclinical studies in rheumatoid arthritis have shown promising results by antagonizing TLR2 and TLR4, targeting their endogenous ligands, reducing their synovial expression levels, and inactivating TLR3, TLR7, TLR8, and TLR9 [179]. TLRs are also implicated in neuroimmune diseases, including multiple sclerosis and Guillain-Barré syndrome, serving as potential therapeutic targets [180]. TLR ligands are currently being investigated in clinical trials for the treatment of various cancer types [39]. Furthermore, the downstream pathways of TLRs are also being explored as potential targets for treating various diseases. IRAK4 inhibitors have completed early-stage trials for the treatment of rheumatoid arthritis and systemic lupus erythematosus [38].

Since TLRs have such a determining role in the pathogenesis of AD, they could potentially serve as great drug targets. Several regimens currently used for AD exert their effects partly through TLRs. Topical vitamin-D analogs and topical tacrolimus upregulate TLR2 expression [[181], [182], [183], [184]]. Cyclosporine, as a systemic calcineurin inhibitor, increases the responsiveness of keratinocytes to TLR2 [185,186]. Histamine H4 receptor (H4R) antagonists can inhibit TLR2-mediated upregulation of chemokines that promote Th2 response [101]. Furthermore, phototherapy-induced immunosuppression is dependent on TLR3 and TLR4 [127,187].

Direct stimulation of TLRs has shown therapeutic benefits. The topical application of 5 % *V.filiformis* lysate cream (TLR2 ligand) improved symptoms in patients with AD [165,166]. Application of γPGA (TLR4 ligand) reduces AD symptoms, basophil population, and Th2 cytokines levels and upregulates Th1 cytokines through the TLR4/DC/IL12 axis in mice models of AD [158]. A trypsin hydrolysate obtained from *Tenebrio molitor* mitigated both serological and histological symptoms of AD in mice through the inhibition of the TLR2/MyD88-dependent pathway [188]. TLR9 agonists such as CpG ODN have shown promising results in the treatment of canine and mice models of AD, cutaneous allergen-induced immunization, and Th2-dominated inflammatory diseases [153,155,189,190]. On the contrary, restraining intracellular signaling pathways, including TLR4/MyD88/NF-κB, conjugated linoleic acid (CLA), a mixture of fatty acids abundant in dairy products and beef, lowers AD symptoms, inflammatory cells, cytokines, and antibodies [191]. Likewise, modulating TLR8 expression with miRNA through the Salmonella vector improves symptoms and cytokine milieu in AD mouse models [41]. Several herbal agents, including Calycocin and Osthole, improve AD characteristics through inhibition of TLR pathways in vitro and in mice AD models [186,[192], [193], [194]].

Utilizing TLRs as therapeutic targets in AD presents several significant challenges. The ubiquitous expression of TLRs across multiple cell types and tissues raises concerns about off-target effects and systemic immune responses [195]. The multifaceted role of TLRs in AD - promoting both Th1 and Th2 responses depending on the context, impairing pathogen clearance, and driving excessive inflammation - renders precise therapeutic modulation particularly challenging. Dosing optimization remains challenging, as excessive TLR activation could potentially exacerbate inflammation, while insufficient stimulation might fail to achieve therapeutic benefits.

Heterogeneity in TLR expression levels, polymorphisms, and TLR immune responses complicates the development of standardized treatment protocols. There are inconsistencies in the expression patterns and functions of TLRs in AD (Table 1). Many factors, including heterodimerization, interactions with other macromolecules and receptors and intrinsic TLRs alterations due to genetic polymorphisms, can influence both the expression pattern and function of TLRs (Table 2). Furthermore, the disease stage and skin condition can affect TLR expression levels and functions. TLR2 levels in monocytes were found to be increased during the exacerbation period but not in the 4-month follow-up [84]. Th1 cytokines, which are abundant during the chronification phase of the disease, increase the TLRs expression [196]. The differentiation levels of sampled cells may affect the expression levels as it has been shown that highly differentiated keratinocytes express less TLR2 [49]. Similarly, physical skin injury can upregulate TLR2 expression [97]. Also, cytokine milieu has a determining role in TLR functions as it has been shown that upon TLR2 stimulation, IL-4 converts a self-limited Th2 response to a perpetuating inflammation through downregulation of IL-10 and upregulation of IFN-γ and IL-12 ^105,106^. Furthermore, the prevailing bacterial colonization, alteration in the structure of glycoproteins and ligands, relative frequency of and affinity of each TLR, could also affect TLR functions. Thus, in AD, the stimulation of TLRs could have very diverse consequences ranging from immunomodulatory to proinflammatory Th1/Th2 dominant responses. Future studies might offer a greater understanding of the role of TLRs in AD.

## 4 Future research directions

Future research on TLRs in AD pathogenesis should focus on several promising directions. CRISPR (Clustered Regularly Interspaced Short Palindromic Repeats) is a gene-editing technology derived from bacterial defense mechanisms against viral infections [197]. The CRISPR system, typically utilizing the Cas9 enzyme, allows precise DNA modifications by directing molecular 'scissors' to specific genetic sequences, enabling the addition, removal, or alteration of DNA, thus providing new insights into gene functions and facilitating targeted manipulation [198]. By using CRISPR to modify the function of TLR genes and their downstream pathways in various contexts, including AD and specific cell lineages, the role of TLR genes within these environments can be better elucidated [199]. Furthermore, CRISPR-based modulation of TLR pathways offers significant potential for developing novel immunotherapies and enhancing vaccine adjuvants [200]. Multi-omic studies aimed at identifying novel SNPs, transcriptoms, downstream components of TLR pathways implicated in AD pathogenesis, and the effects of different treatments on these elements could further enhance our understanding of the role of TLRs in AD [201].

Development of more specific TLR agonists and antagonists holds the potential to significantly improve therapeutic strategies by precisely modulating TLR activity. Targeting specific TLRs involved in disease processes could lead to treatments that more effectively regulate immune responses without broadly suppressing the immune system, thereby reducing the risk of adverse effects. This precision could enhance the efficacy of therapies by focusing on key pathways involved in AD and other inflammatory conditions while minimizing unwanted side effects, making treatments safer and more tolerable for patients over the long term. Furthermore, identifying new ligands in the context of AD could help explain the discrepancies observed in the literature and propose new therapeutic options to target TLR function. Incorporating personalized medicine approaches to tailor TLR-targeted therapies based on individual genetic profiles, such as the presence or absence of known SNPs associated with AD, could allow for customized treatments that align with the patient's unique immune profile, potentially improving efficacy, minimizing side effects, and enhancing overall therapeutic outcomes (Table 2) [202,203].

Machine learning (ML) methods, including deep learning, could be used to improve patient outcomes in AD by enhancing our understanding of TLRs. ML methods could be utilized to discover novel TLR modulators by analyzing structure-activity relationships and predicting molecular interactions [204]. Methods including random forests have been used to identify new TLR ligands [205,206]. Additionally, ML methods could be used to directly assess the risk and prognosis of AD based on factors such as the expression levels of TLRs and the presence of polymorphisms (Table 1, Table 2). These predictive models may help identify which patients would benefit most from specific TLR agonists or antagonists, enabling personalized immunomodulatory strategies.

Conducting studies across more diverse settings, in terms of factors such as disease stage, genetic profiles, cell lineages, and other variables, can help clarify the specific factors influencing TLRs in AD and address the discrepancies noted.

## 5 Conclusion

The function and expression patterns of TLRs are altered in AD. The intricate involvement of TLRs in barrier dysfunction and immune dysregulation represents a critical therapeutic target in AD. Subsequently, the development of targeted TLR modulators must carefully balance immune activation and suppression to achieve optimal clinical outcomes. Successful therapeutic strategies will likely require personalized approaches that account for individual variations in TLR expression patterns and functional responses, ultimately leading to more effective treatments for this challenging condition.

## CRediT authorship contribution statement

**Ahmad Vafaeian:** Writing – review & editing, Writing – original draft, Visualization, Validation, Project administration, Methodology, Investigation, Data curation, Conceptualization. **Fateme Rajabi:** Writing – review & editing, Visualization, Validation, Project administration, Methodology, Investigation, Conceptualization. **Nima Rezaei:** Writing – review & editing, Visualization, Validation, Supervision, Project administration, Methodology, Investigation, Conceptualization.

## Disclosures

None.

## Ethics statement

Not applicable.

## Ethical approval

Due to the utilization of data from already published studies, obtaining ethical approval is unnecessary for this study.

## Data and code availability statement

No new data was generated for the research described in the article.

## Funding statement

This research received no specific grant from any funding agency in the public, commercial, or not-for-profit sectors.

## Declaration of competing interest

The authors declare that they have no known competing financial interests or personal relationships that could have appeared to influence the work reported in this paper.
